# Mapping the Lobbying Footprint of Harmful Industries: 23 Years of Data From OpenSecrets

**DOI:** 10.1111/1468-0009.12686

**Published:** 2024-01-14

**Authors:** HOLLY CHUNG, KATHERINE CULLERTON, JENNIFER LACY‐NICHOLS

**Affiliations:** ^1^ Centre for Health Policy, Melbourne School of Population and Global Health The University of Melbourne; ^2^ School of Public Health The University of Queensland

**Keywords:** corporate political activity, lobbying, United States, harmful industries, commercial determinants of health

## Abstract

**Policy Points**

- Our research reveals the similarities and differences among the lobbying activities of tobacco, alcohol, gambling, and ultraprocessed food industries, which are often a barrier to the implementation of public health policies.
- Over 23 years, we found that just six organizations dominated lobbying expenses in the tobacco and alcohol sectors, whereas the gambling sector outsourced most of their lobbying to professional firms.
- Databases like OpenSecrets are a useful resource to monitor the commercial determinants of health.

**Context:**

Commercial lobbying is often a barrier to the development and implementation of public health policies. Yet, little is known about the similarities and differences in the lobbying practices of different industry sectors or types of commercial actors. This study compares the lobbying practices of four industry sectors that have been the focus of much public health research and advocacy: tobacco, alcohol, gambling, and ultraprocessed foods.

**Methods:**

Data on lobbying expenditures and lobbyist backgrounds were sourced from the OpenSecrets database, which monitors lobbying in the United States. Lobbying expenditure data were analyzed for the 1998–2020 period. We classified commercial actors as companies or trade associations. We used Power BI software to link, analyze, and visualize data sets.

**Findings:**

We found that the ultraprocessed food industry spent the most on lobbying ($1.15 billion), followed by gambling ($817 million), tobacco ($755 million), and alcohol ($541 million). Overall, companies were more active than trade associations, with associations being least active in the tobacco industry. Spending was often highly concentrated, with two organizations accounting for almost 60% of tobacco spending and four organizations accounting for more than half of alcohol spending. Lobbyists that had formerly worked in government were mainly employed by third‐party lobby firms.

**Conclusions:**

Our study shows how comparing the lobbying practices of different industry sectors offers a deeper appreciation of the diversity and similarities of commercial actors. Understanding these patterns can help public health actors to develop effective counterstrategies.

The definition of commercial determinants of health (cdohs) set out in *The Lancet* 2023 series recognizes that commercial actors are diverse and have different impacts on health.^1^ Yet too often, public health advocates fail to make these distinctions, referring to “the industry” or “corporations” as a proxy for harmful commercial actors.^2^ This lack of nuance stymies efforts to develop a science of commercial determinants.

One way to start thinking through the differences among commercial actors is to compare the practices and attributes of different types of actors.^2^, ^3^ In this study, we contrast the lobbying activities of four industry sectors that severely impact health and have been the focus of much public health research and advocacy: tobacco, alcohol, gambling, and ultraprocessed food (UPF) companies. In addition to comparing commercial actors based on their portfolio, we also differentiate between individual companies and industry trade associations, a distinction that is often missing in empirical studies of political activity.^4^ Beyond this paper's conceptual focus on commercial actor diversity, it also seeks to investigate which characteristics of commercial lobbying are feasible to capture at scale. In so doing, it supports efforts to systematically monitor the CDoHs.

Attention to CDoHs has grown in recent years, with the World Health Organization (WHO)’s launch of a new program of work in 2021 and the launch of *The Lancet* series on CDoHs offering two prominent illustrations.^1^, ^5^ Within this emerging discipline, there are many streams of work analyzing different aspects of CDoHs, including system dynamics such as neoliberalism and capitalism^1^, ^6^; the diversity of commercial actors^2^, ^7^; commercial practices influencing science, marketing, and politics^8^, ^9^, ^10^; and the myriad of case studies concerning harmful industry sectors such as alcohol, gambling, tobacco, UPFs, guns, and fossil fuels.^11^, ^12^

This study focuses on one commercial activity: lobbying. Lobbying is one of several political strategies that commercial actors use to influence policymaking.^8^ Evidence demonstrates that countries with a greater degree of corporate permeation are less likely to implement evidence‐based health policies endorsed by the WHO,^13^ and more recently, lobbying practices by companies and trade associations have been linked to efforts to influence US participation in and funding of WHO.^14^ Although the tobacco industry has a long history of lobbying to deliberately stall, weaken, and block public health regulations of its industry, there is growing evidence that the same political practices are used by gambling, alcohol, and UPF industries to oppose policies that threaten their business interests.^15^, ^16^, ^17^, ^18^ We note that much of this research relies on documentary analysis, as empirical data sets of lobbying are rare.^19^, ^20^ Analyzing and monitoring political practices like lobbying is challenging, as information about commercial lobbying and political donations is often poorly disclosed, delayed, or lacking relevant information.^21^ In this study, we aimed to explore the utility of one notable non‐governmental organization (NGO) database (OpenSecrets) to monitor corporate lobbying. This study is part of our broader program to explore approaches to monitor CDoHs.

Lobbying is defined in different ways, with an Organisation for Economic Co‐operation and Development survey noting that no country used the same definition.^22^ In 2022, a number of NGOs developed the International Standards for Lobbying Regulation, which defined lobbying as “any direct or indirect communication with a public official that is made, managed, or directed with the purpose of influencing public decision making.”^23^ Many different activities have been conceptualized as a form of lobbying, including meeting with public servants, coordinating public campaigns to influence voters, funding astroturf organizations (designed to appear as genuine grassroots advocacy), or submissions to policy processes.^20^ In some cases, companies have staff employed in‐house to specifically focus on lobbying (e.g., government relations units). In other cases, companies hire third‐party (external) lobby firms to lobby on their behalf. Although concerns have been raised about the undue influence of some businesses and industry sectors in politics, it is important to note that lobbying itself is a legitimate practice in democratic governments and can support representative policymaking.^23^, ^24^

Lobbying can be a resource intensive activity, with an estimated US $4.1 billion spent on lobbying in the United State in 2022.^25^ This makes it easier for well‐resourced organizations (like many businesses and trade associations) to engage in lobbying and other political activities.^26^ Lobbying resources can go beyond the money spent hiring lobby firms (or employing lobbyists directly). One longer‐term strategy that can increase the effectiveness of lobbying is the revolving door, which is the movement of individuals from employment in government in political or administrative roles (e.g., elected officials or civil servants) to private industry, and vice versa.^27^ Revolving‐door practices are particularly common for third‐party lobbyists and are understood to confer three main categories of benefits to the lobbyist and their clients: they can leverage professional networks to achieve their clients’ goals; they have intimate knowledge of governmental processes, which can inform strategies; and they may also have insider knowledge regarding government preferences concerning specific policy matters.^27^, ^28^, ^29^ In the absence of enforced cooling‐off periods after exiting public office, the revolving door can also present risks for conflicts of interest to arise, especially if the former government employee moves into a lobbying role that focuses on their former portfolio.^30^

Efforts to systematically monitor lobbying face several challenges. No single measure of lobbying is consistently available internationally, presenting challenges for efforts to develop global indices of corporate political activity or CDoHs.^13^, ^31^ Of the three monitoring frameworks proposed to measure the influence of commercial actors, lobbying only appears as an indicator in one: the CDoHs index.^31^ Authors of the Corporate Permeation Index and Corporate Financial Influence Index excluded lobbying indicators, as there were insufficient comparable data on this metric across countries, though the authors acknowledged lobbying as an important mechanism of CDoH influence on policymaking.^32^, ^33^ Alongside academic efforts to monitor lobbying and corporate political activity are the civil society groups and NGOs who play an active role in monitoring commercial practices, drawing public and policymaker attention to commercial harms and advocating for transformative changes so that people are prioritized over profits.^34^, ^35^

Some NGOs have developed databases for monitoring and sharing information about commercial lobbying. OpenSecrets is a nonprofit organization that maintains one of the most extensive databases on political donations, lobbying expenditure, and revolving‐door practices in the United States.^36^ This database presents an opportunity to monitor corporate lobbying over time and to compare the practices of different industry sectors. This study seeks to expand our understanding of the corporate political activities of four industries that profoundly affect health (tobacco, alcohol, gambling, and UPFs). By exploring one of the more complete data sets concerning corporate lobbying, we aim to answer two questions. First, which patterns could be identified about how different commercial actors engage in lobbying over time? Second, what are the opportunities and limitations afforded by the OpenSecrets database? In the discussion, we reflect on our learnings as well as some of the challenges we faced. We conclude by proposing ways that this database could be adapted and modified so that other jurisdictions can more easily monitor corporate political activity.

## Methods

This project utilized secondary data from the US‐based OpenSecrets database. OpenSecrets is a publicly accessible online database that is primarily used to monitor political activity in the United States—this includes campaign financing, lobbying expenditure, and the revolving door.^36^ Although many of the political activities target domestic laws, there are examples of lobbying to impact foreign policies (e.g., US policies toward the WHO).^14^ The lobbying database, accessible from OpenSecrets, is compiled from reports filed with the Senate Office of Public Records (SOPR) in accordance with the Lobbying Disclosure Act.^36^ Mandatory reports are filed by organizations and lobby firms each quarter and uploaded to the SOPR website, where they are then retrieved by OpenSecrets.^37^ OpenSecrets then transforms these reports into an interoperable format. As part of its additional analysis, OpenSecrets links these records with its revolving‐door database of lobbyists’ former government employment. OpenSecrets has also developed an industry classification framework and assigns each organization to one of those categories. The OpenSecrets databases uses the term “client” to refer to the companies and trade associations that lobby (including those that employ all their lobbyists in‐house), and we use that term throughout the results.

### Data Collection

Data collection and analysis occurred in two phases. In the first phase, annual lobbying expenses were collected for the period 1998–2020. For this study, we focused on five OpenSecrets designated categories that best represented the tobacco, alcohol, gambling, and UPF sectors (tobacco; casinos/gambling; beer, wine, and liquor; and food and beverage and food processing and sales). We note that many companies in these categories have diverse portfolios (for instance, Coca‐Cola has a large bottled‐water segment) and that not all products are universally or equivalently harmful to health. We reflect on this limitation in the discussion. Although OpenSecrets provides the option of accessing its bulk data on request, despite several requests, we were unable to access the bulk data and instead relied on manually downloading individual spreadsheets. Individual comma‐separated value (CSV) files (*n* = 110) were downloaded from the OpenSecrets website between May 15, 2022, and June 22, 2022. These files contained the lobbying expenses for all clients that were classified as part of that industry sector. All expenditure data were adjusted for inflation using gross domestic product price deflators sourced from the World Bank Databank.^38^ All results are reported in 2020 values.

We reviewed all individual clients and coded them as either commercial company or trade association. Clients were categorized based on researcher knowledge and confirmed through conducting web searches of the client's name to find additional information. Many of the clients lobbying in the gambling sector were Native American nations where casinos were located. We coded these clients as companies, as most were incorporated entities.

To gain a greater understanding of types of individual lobbyists that clients employed, we collected data on the lobbyists employed by the top two companies and trade associations for each sector (measured in terms of their net expenditure between 1998 and 2020). All files detailing lobbyists employed by these 16 clients (*n* = 333) were extracted from the OpenSecrets database between July 22, 2022, and July 26, 2022.

The OpenSecrets database separates the lobbying expenses for in‐house lobbyists (directly employed by the company or trade association) and third‐party lobbyists (employed by external lobby firms). Although we can see the total amount spent for each lobby firm as well as the specific lobbyists that lobbied on behalf on the client, lobbying expenses were not disaggregated to the level of individual lobbyists (rather the total amount for the lobby firm is listed against each lobbyist). To avoid double counting, we divided this number by the number of lobbyists active in that year (i.e., if a firm spent $100,000 and employed four lobbyists, we assigned each lobbyist a $25,000 value).

### Data Cleaning and Analysis

One of the challenges of analyzing commercial activity over time is that companies change the products they sell, their ownership, and their name. Some companies have diverse portfolios, making it challenging to establish a single industry classification.

We first reviewed changes in client ownership and names over time. This was facilitated by the OpenSecrets database, which retroactively linked the spending data of an acquired company to its new parent company. For example, a search for an overview of lobbying expenditure for tobacco company Altria includes data from 1998 to present. However, when the search is limited to the years 1998–2002, the “client profile” is listed as Philip Morris, not Altria (Philip Morris USA changed its name to Altria in 2003, and the company has gone through several changes in ownership and structure).^39^ Essentially, the lobbying expenditures that businesses incurred before they were acquired is added to their parent company's total expenditure. Although this allows us to get a sense of the history of a company's lobbying, it also means that historic records must be manually reviewed to match companies with their current parent company. To identify linked companies, we manually reviewed each client in our original data set (*n* = 1,047) for similar names and triangulated this with searches on the OpenSecrets database and Google to confirm the current name. Although we have done our best to be complete, it is possible that we have missed some connections, and we discuss opportunities to address this data‐cleaning challenge in our discussion.

We also reviewed all clients in the data set (*n* = 1,047) and assigned each client to a single industry classification. Although we mainly relied on the classification and naming scheme applied by the OpenSecrets database, in nine cases, a company or trade association was classified to more than one sector. For example, the OpenSecrets database classified alcohol company Diageo, PLC, as both food and drink (1998‐2000, $6,161,728 total) and alcohol company (1998‐2020, $48,860,519 total). This reflects the company's portfolio diversity at the time (for instance, in 1998, Diageo owned Burger King among other companies). Similarly, tobacco company Altria was classified as a food‐processing company from 2003 to 2006 and as a tobacco company from 2004 to 2020. To determine an overall classification for these nine clients, the total lobbying expenses for the client were reviewed, and we coded them to the sector with the greatest lobbying expenses.

To explore whether market share helped to explain lobbying activity, we matched the companies in the OpenSecrets database to Euromonitor market share data. We identified 12 relevant Euromonitor market share data sets for alcohol (alcoholic drinks), UPFs (snacks, soft drinks, staple foods), and tobacco industries (cigarettes, cigars and cigarillos, e‐vapor products, heated tobacco, smokeless tobacco, smoking tobacco, tobacco‐free oral nicotine, tobacco heating devices). (Euromonitor does not provide market share data about the gambling sector in the United States.) Although some data sets covered a longer period, all covered the years 2013–2020 (though for several of “vapor” tobacco products, there were no data in earlier years). For each data set, we documented the 20 companies with the largest market share for each year (several of the tobacco data sets only had a few companies). For the years 2013–2020, we identified 25 alcohol companies, 70 UPF companies, and 26 tobacco companies. We then searched for these companies in the OpenSecrets data using Excel's Fuzzy Lookup function to create a matched list (i.e., coding “Coca‐Cola Co, The” in the Euromonitor data to “COCA‐COLA CO” in the OpenSecrets data). We manually reviewed all matches.

We used Power BI software to create a relational data model to link the tables for analysis and visualization. Our findings are presented below.

## Results

Between 1998 and 2020, the alcohol, gambling, UPF, and tobacco industry sectors spent approximately 3.26 billion USD on lobbying in the United States (adjusted to 2020 values). This comprises just 5.7% of total lobbying expenditure in the United States for this time period (approximately 56.7 billion USD). The UPF industry spent the most ($1.15 billion), followed by gambling ($817 million), tobacco ($755 million), and alcohol ($541 million).

**Figure 1:**
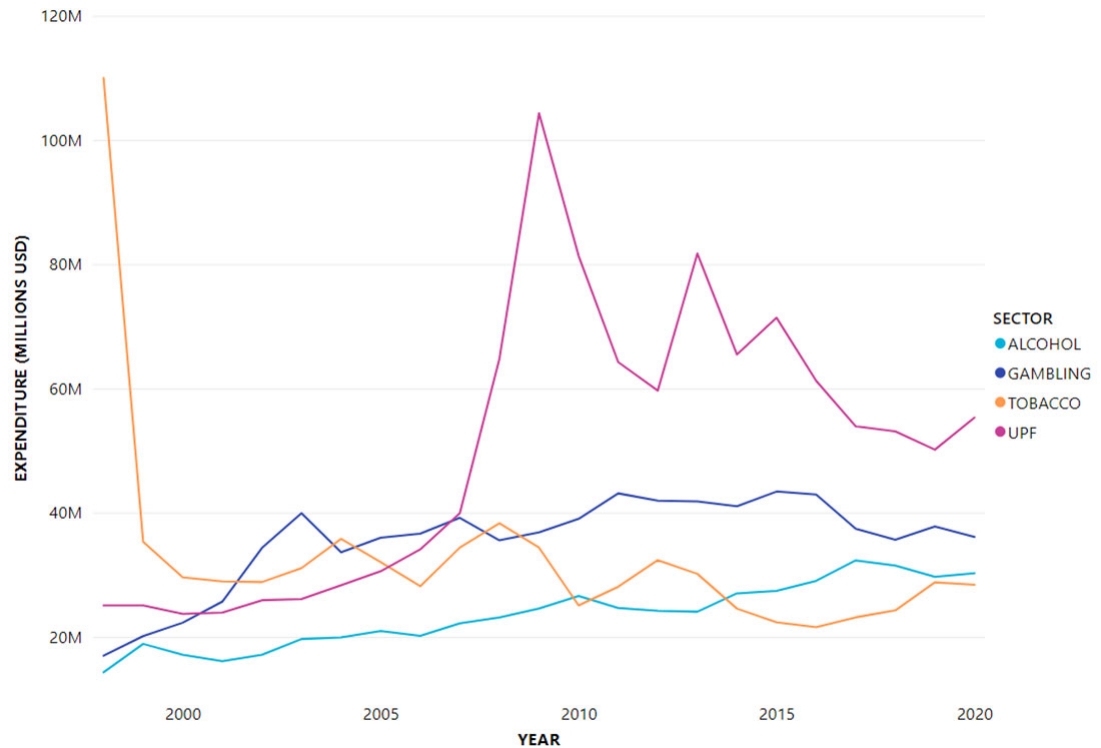
Lobbying Expenditure by Sector 1998–2020 [Colour figure can be viewed at wileyonlinelibrary.com] UPF, ultraprocessed food.

Spending over time has varied for each sector (Figure 1). Tobacco expenditure peaked at over US $110 million in 1998, dropping to just over US $35 million in 1999. Since then, its spending has ranged between 20 and 40 million USD annually. Alcohol spending was the lowest in 1998, and has steadily increased over time, but remains low overall relative to the other sectors (its highest expense was $32 million in 2017). Gambling expenditure more than doubled between 1998 and 2003 and has ranged between $35 and $45 million since then. UPF spending peaked in 2009 ($104 million) and has decreased since then, with smaller peaks in 2013 and 2015.

Differences in lobbying spending could be observed within each sector. Although companies spent more than trade associations on lobbying overall, comprising 77% of all spending on average, this ratio varied by sector. Trade associations were most active in the alcohol industry (43% of total expenditure), followed by UPF associations (32%), gambling (14%), and tobacco (5%) (Figure 1).

**Figure 2:**
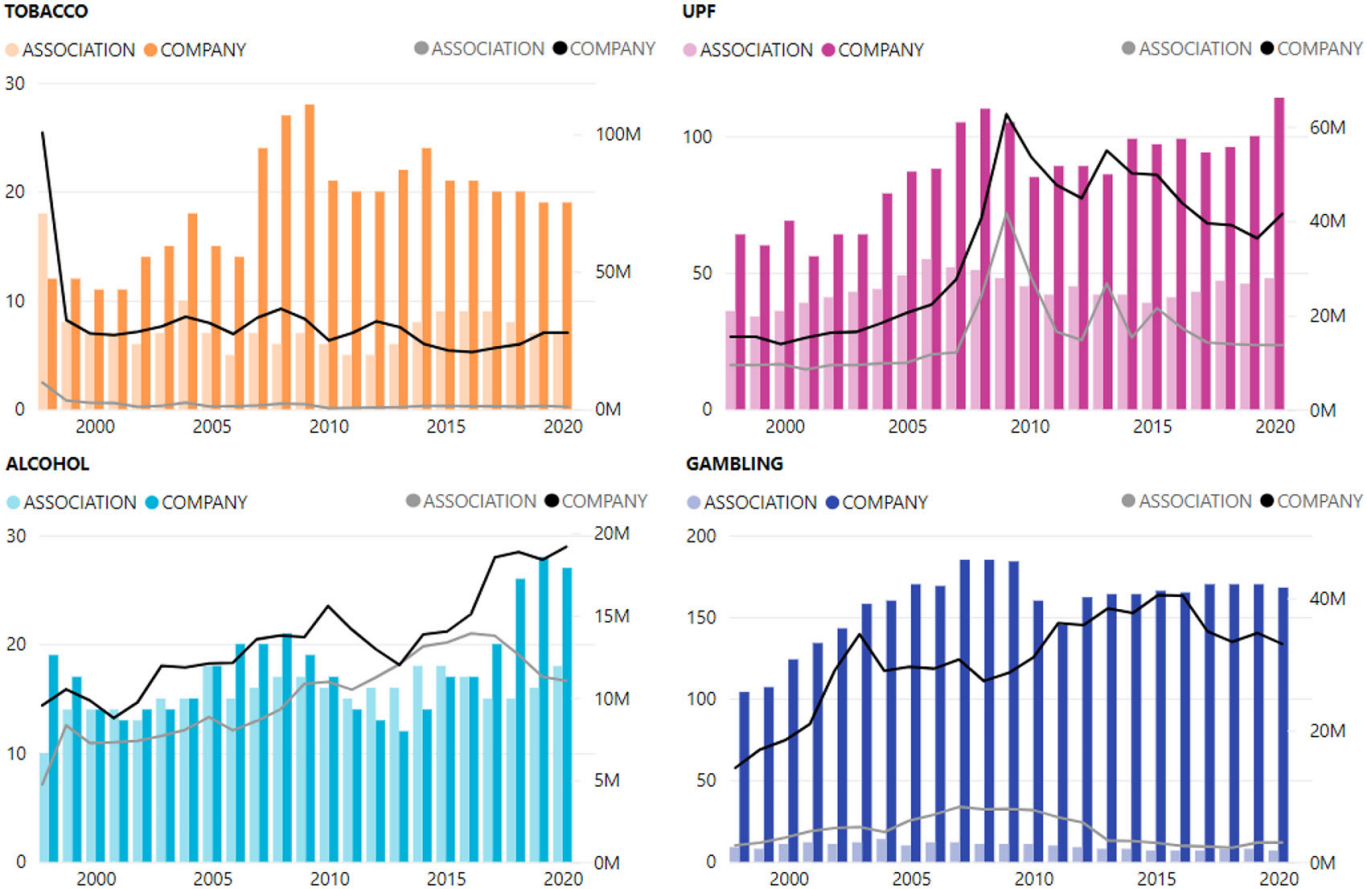
Comparison of Company and Trade Association Lobbying 1998–2020 [Colour figure can be viewed at wileyonlinelibrary.com] Bars represent the count of clients and associations active each year, with values shown on the y‐axis. Lines represent the amount spent on lobbying (millions USD), with values shown on the secondary y‐axis (right side). The x‐axis shows the year. UPF, ultraprocessed food.

The number of companies and associations engaged in lobbying differed across sectors and over time. The UPF industry had the highest number of unique clients engaged in lobbying between 1998 and 2020 (*n* = 462), followed by gambling (*n* = 351), alcohol (*n* = 109), and tobacco (*n* = 86). For all sectors, the number of clients engaged in lobbying surged between 2007 and 2009, after which tobacco and gambling clients decreased, whereas alcohol and UPF clients increased. There was not always a correlation between the number of clients engaged in lobbying and the total amount of money spent (Figure 2).

**Figure 3:**
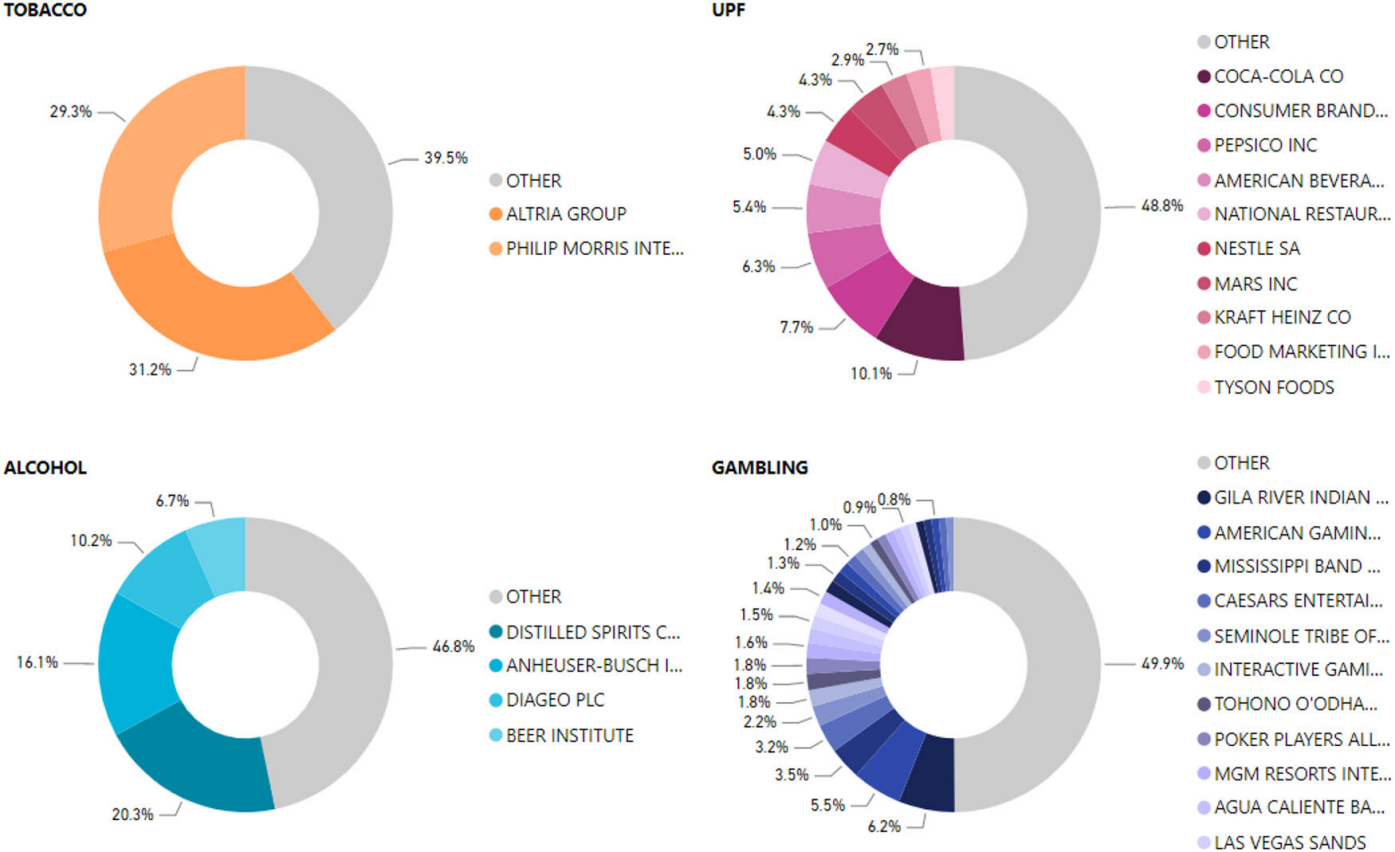
Concentration of Lobbying Spending by Key Clients [Colour figure can be viewed at wileyonlinelibrary.com] UPF, ultraprocessed food.

Within each sector, we also explored which clients had the highest expenditures and whether this was stable over time (Figure 3). The tobacco industry displayed the highest degree of concentration, with two clients accounting for over 60% of all spending (Altria Group, 31%; Philip Morris International, 29%). The other sectors displayed less concentration, with the top 50% of spending spread across four clients in alcohol, 10% in UPFs, and 30% in the gambling sector.

**Figure 4:**
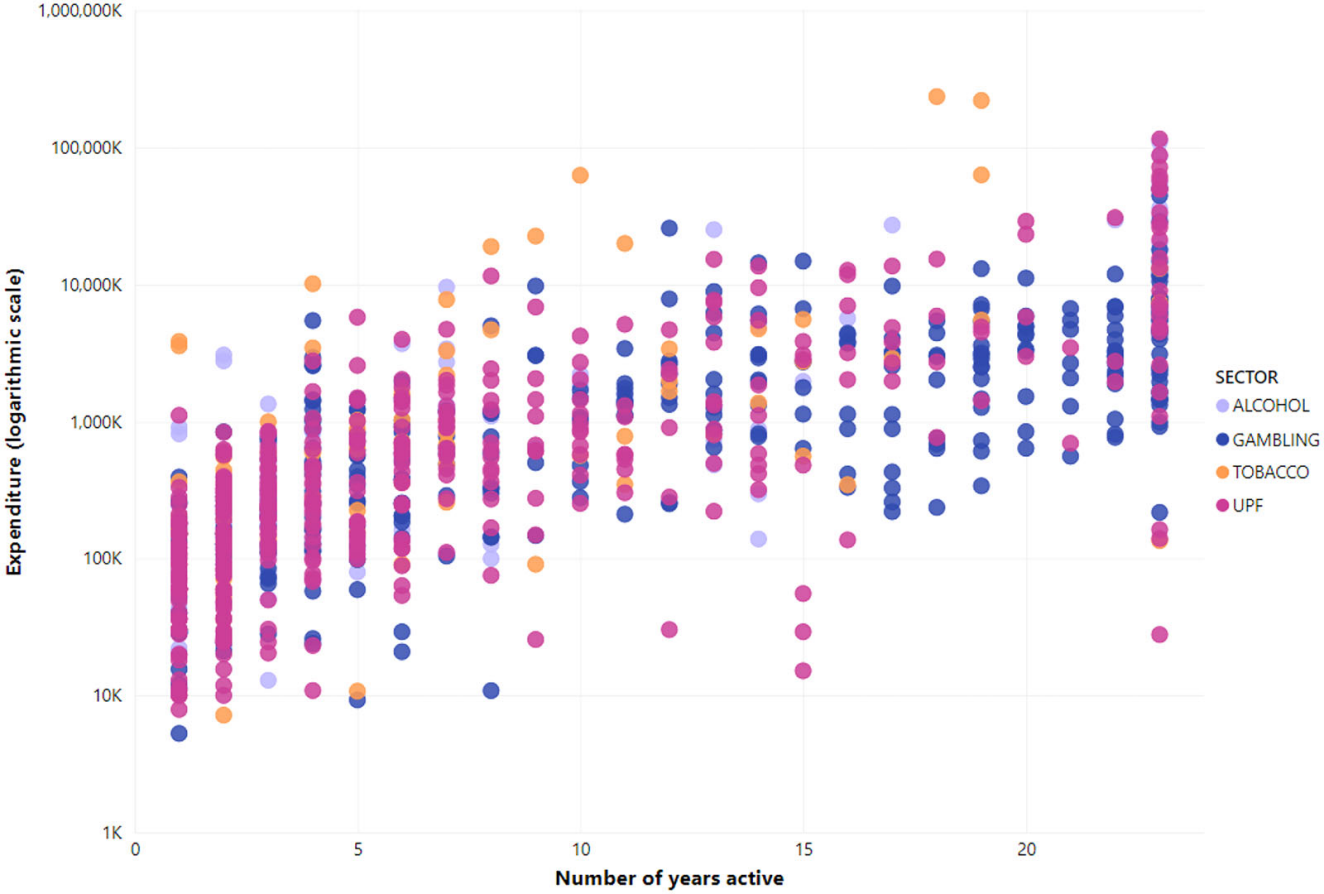
Years Active Compared With Overall Spending [Colour figure can be viewed at wileyonlinelibrary.com] UPF, ultraprocessed food.

There was some correlation between the amount of money a client spent on lobbying and the number of years they were active. However, some clients only lobbied in 1 year but spent far more than others that lobbied every year (for example, the Tobacco Institute lobbied once, spending $3.6 million dollars [it was disbanded in 1998], compared with Seafreeze, LTD, which lobbied every year but spent only $27,779 overall). This variation can be seen in Figure 4, which shows clients with higher spending and fewer years of lobbying (upper left section), compared with clients with lower spending but active most years (lower right section).

**Figure 5:**
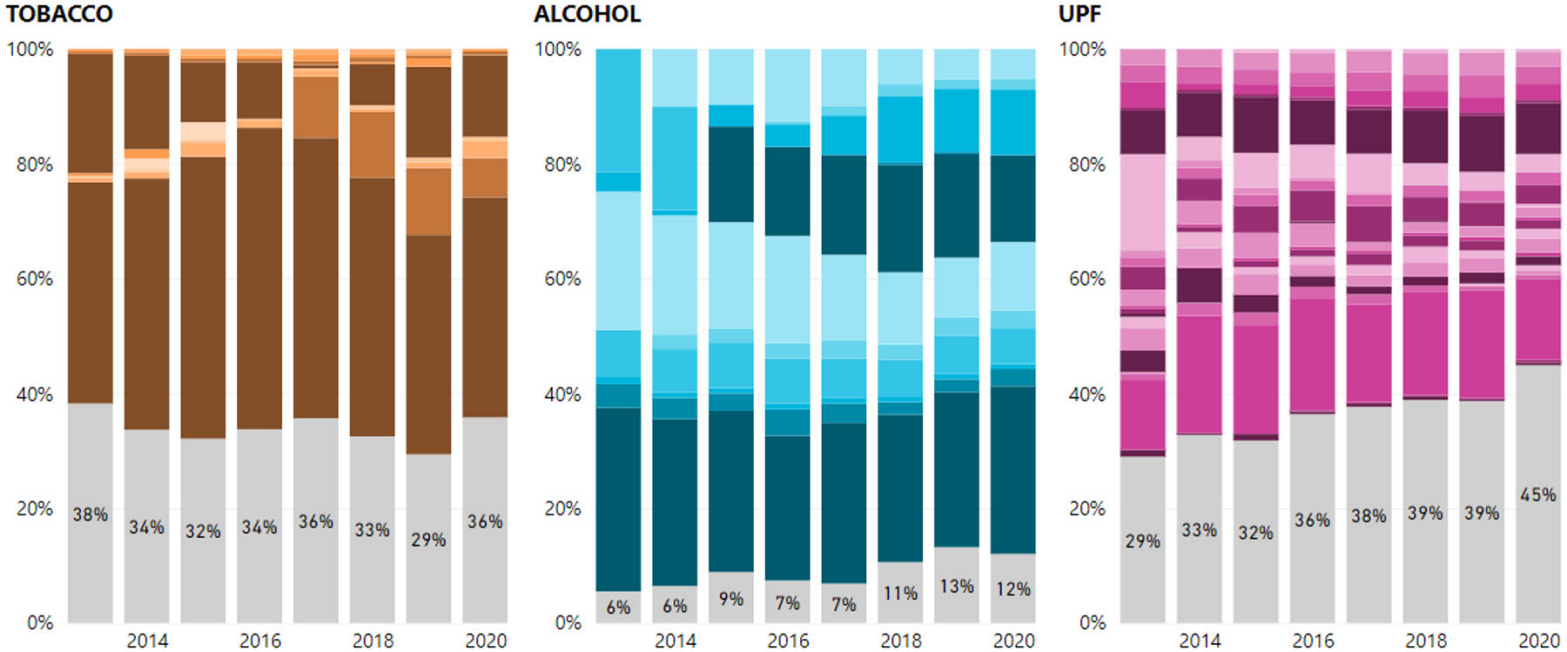
Percentage of Lobbying Expenditure From Largest Market Share Clients [Colour figure can be viewed at wileyonlinelibrary.com] UPF, ultraprocessed food.

Of the companies with the largest market share in the tobacco, alcohol, and UPF sectors, only a portion were documented as spending money lobbying in the OpenSecrets database: 15 (60%) of 25 alcohol companies, 14 (54%) of 26 tobacco industry companies, and 36 (51%) of 70 UPF companies lobbied. However, these companies accounted for a substantial percentage of total company lobbying expenditure. Between 2013 and 2020 (the years for which we had market share data), the largest market share companies accounted for approximately 66% of tobacco company lobbying expenses, 90% of alcohol company lobbying expenses, and 64% of UPF company lobbying expenses, though this varied year to year (Figure 5).

**Figure 6:**
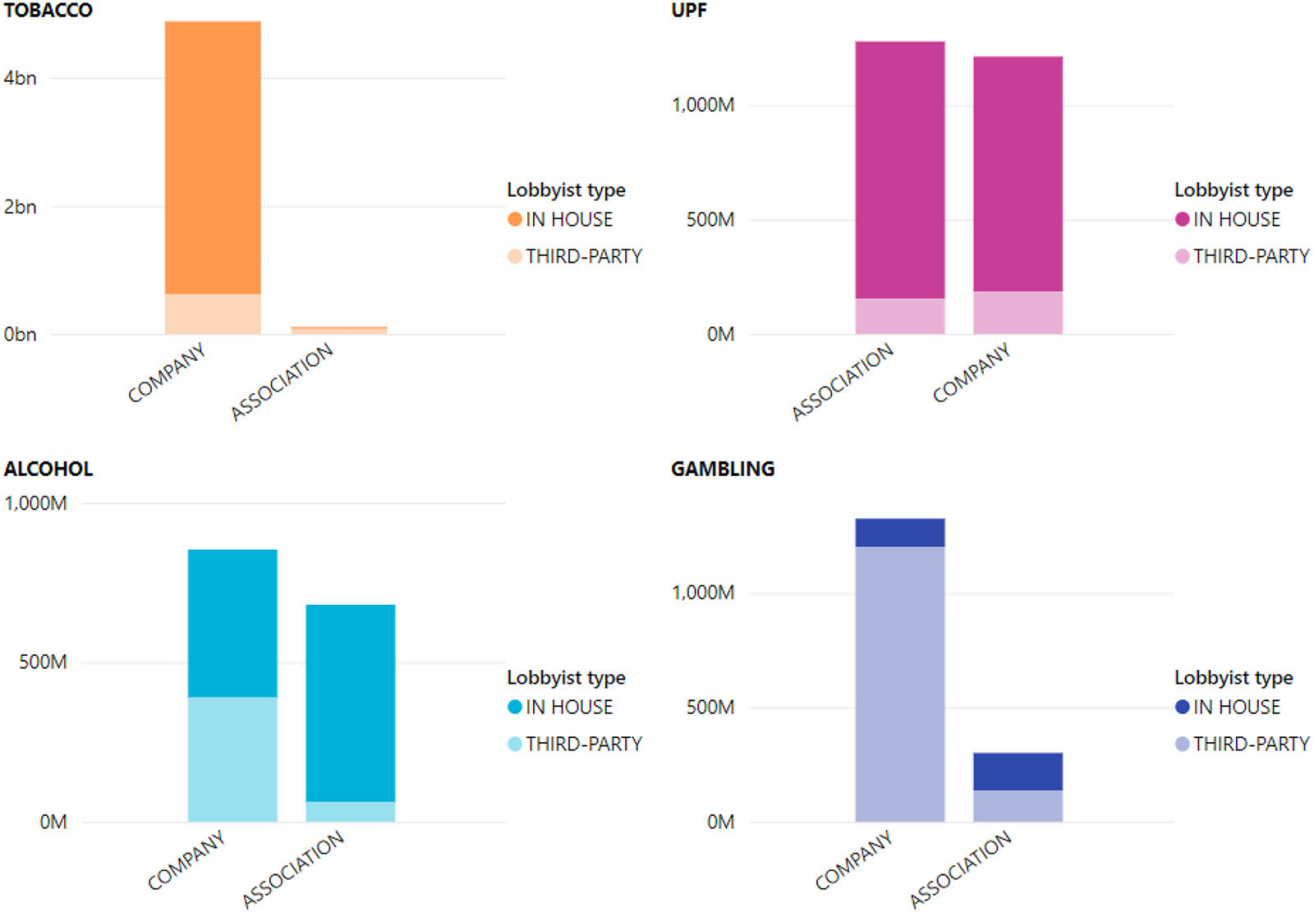
Lobbying Spending on In‐House Versus Third‐Party Lobbyists [Colour figure can be viewed at wileyonlinelibrary.com] UPF, ultraprocessed food.

The second phase of our study explored the lobbying practices of the top two companies and trade associations for each industry sector with the largest overall lobbying expenditure (*n* = 16 clients). We first analyzed whether each client preferred in‐house or third‐party lobbyists. When comparing the amount spent on each category of lobbyist, the tobacco and UPF industries had the highest ratio of spending allocated to in‐house lobbyists (both 86%), followed by alcohol (71%), whereas gambling only allocated 18% to in‐house lobbyists. When we compared companies and trade associations, only two groups spent more on third‐party lobbyists: tobacco associations and gambling companies. All others spent more on in‐house lobbyists (Figure 6). However, when analyzing the number of lobbyists hired, all industries hired far more third‐party lobbyists overall, despite spending more on their in‐house lobbyists, with third‐party lobbyists comprising 95% of all gambling industry lobbyists, followed by tobacco (91%), alcohol (87%), and UPF (78%).

We also examined the background of the lobbyists. The OpenSecrets website has a revolving‐door database with information on the previous government employment of many lobbyists in the database. Overall, 62% of lobbyists were former government employees, 3% were former members of Congress (Senate and/or House of Representatives), and 35% did not have a government background (according to the OpenSecrets database). This ratio remained similar when we compared industries (with tobacco using slightly fewer revolving‐door lobbyists), as well as when comparing lobbyists used by companies and trade associations. Differences emerged when we compared in‐house versus third‐party lobbyists, with far fewer in‐house lobbyists having a background in government (35% compared with 71% for third‐party lobbyists). Almost all lobbyists that were former members of Congress worked for a third‐party lobby firm (*n* = 40), with only three former members of Congress employed in‐house (two employed by a food industry association and one by an alcohol association).

## Discussion

The OpenSecrets database allows tracking of lobbying expenditure over time. This can be helpful for public health advocates to understand where different industries are focusing their efforts and which issues they are actively trying to stop progressing. For example, we can see that certain peaks in spending correspond with various policy issues. In 2009, when a tax on sugary drinks was proposed, there was a spike in lobbying by the UPF sector (in particular, sugary drink manufacturers and their peak body the American Beverages Association, which itself spent more than $22 million dollars lobbying that year). UPF lobbying expenses were likewise high between 2013 and 2015, when food companies opposed regulations for labeling of genetically modified foods.^40^ Future studies could analyze the OpenSecrets data in relation to the wider political landscape to see if peaks in lobbying correspond with the development of policies or regulations. This could help to get a sense for which policies were of greatest interest to clients as well as who lobbyists chose to meet with during a particular period (e.g., whether the Congress member was on a particular committee).

The variation among active clients provided interesting insights into the lobbying practices of different sectors. Tobacco had only two clients accounting for over 60% of all spending, whereas the gambling sector had over 30%. For those sectors with many different actors, lobbyists face additional challenges with possibly greater fragmentation of policy issues and solutions. This may result in a favorable outcomes for the public health community if the lobbying effort is diluted through fragmentation.^41^ Our study did not analyze this or the specific bills lobbied on by each client (as this was out of scope). However, future studies could identify the number of different clients lobbying on the same bill, whether similar or different positions were taken and the resultant outcome.

Some of our findings seem to counter public health beliefs about corporate political activity. For instance, there is an assumption that the largest companies lobby. However, we found that just over half of the companies with the largest market share were documented as directly undertaking lobbying. It may be that some companies simply do not engage in politics. However, there are many other forms of direct and indirect political advocacy that we did not capture in our analysis, such as political donations or grassroots lobbying, in which those missing companies may be active. Indirect political advocacy (e.g., in which companies fund a think tank or association to lobby on their behalf) is especially opaque. Many of these think tanks and associations are structured as nonprofits and are exempt from financial disclosure requirements in the United States—limiting information about who funds them.^42^ Research suggests that some companies use so‐called “dark money” or hidden forms of advocacy (e.g., lobbying through organizations not subject to disclosure requirements) to avoid reputational damage when supporting causes antithetical to their public positions.^4^, ^42^ Although we did not systematically analyze the membership of the associations in our study, it is likely that many of the companies in the data set (as well as other large companies not in the OpenSecrets database) fund trade associations to lobby on their behalf. Indeed, when we looked at the members of the largest industry associations in our data set, several of their members were also companies that directly funded lobbying. However, many of the member companies did not directly engage in lobbying, instead engaging in lobbying indirectly through their association. This has been seen in the fossil fuel sector, with trade associations taking an active (and sometimes more controversial and oppositional issue) position on climate change issues.^4^, ^43^ So, for some companies, they may lobby via multiple channels: one channel being their own in‐house lobbyists but also via the many trade associations in which they are members.

It is important to recognize that some companies possess considerable lobbying power without having lobbyists employed. Many chief executive officers (CEOs), senior‐level staff, and board members of companies will undertake “lobbying” as part of their day‐to‐day work. These senior executives will often have far greater access to policymakers than professional lobbyists do, as they have inherent power through the significant financial assets they bring to the country. For this reason, policymakers will often prioritize meetings with these individuals.^41^ For those companies who have chosen not to spend on external or in‐house lobbyists, it would be worthwhile investigating the other forms of policy influence they utilize (e.g., meetings with officials by senior executives) to understand their chosen method of influence.

Another finding that challenges public health rhetoric is that we found companies and trade associations were less likely to directly employ lobbyists with government experience. We note that we only examined the lobbyist backgrounds of those hired by 16 of the most active companies and trade associations, so the full data set may tell a different story. However, international studies support our findings, with Canadian studies finding that lobbyists with government experience more often work in consultant roles for third‐party lobby firms rather than in‐house with a company.^28^ An Australian case study of tobacco industry lobbyists also found a slightly higher percentage of third‐party lobbyists had a government background compared with in‐house lobbyists.^44^ One explanation is that lobbyists with extensive political experience and connections are better placed to work across multiple industries and issues and thus work for lobby firms where they have diverse clients.^27^ It is also likely that many of these numbers may be systematically underreported. A US study estimated that half of all lobbyists had worked in government, but only one‐third of them disclosed this in filed Senate records.^45^ Internationally, poor quality or nonexistent lobbying disclosures are an issue for many countries, as most governments provide no lobbying data at all.^19^, ^24^ Last, we also observe that the categories used by OpenSecrets to classify revolving‐door backgrounds are relatively blunt and that a more detailed schema (for instance, highlighting the particular congressional committee or pathway through politics) may offer more insights into why a company may hire a particular lobbyists; e.g., for the access their networks provide and potential conflicts of interest arising from the revolving door.^46^, ^47^

Of significant concern for countries that do not require recording of in‐house lobbyists as part of lobbying disclosure requirements was the finding that most of the sectors used in‐house lobbyists instead of the third‐party firms. This means that many countries are missing most lobbying activities. This urgently needs to be addressed. Similarly, industry trade associations, via their not‐for‐profit status, are sometimes excluded from lobbying regulations.^24^ In light of the substantial amount of lobbying that these organizations provided, especially in the alcohol and UPF sectors, this is another loophole that needs to be urgently reviewed to provide better transparency. Disclosure requirements in many countries could also be strengthened to require a more complete record of meetings with members of Congress, the executive branch, congressional staff, and the bureaucracy. Several governments require detailed contact logs of lobbyist meetings, such as Chile, Ireland, and Canada, though ensuring compliance with the law is an important challenge.

Future studies could develop methods to link lobbying data with political donation data (also captured in the OpenSecrets database). These studies could also expand the scope of industries analyzed to consider other sectors that are heavily regulated, such as the fossil fuel, financial, or property sectors. Some of this analysis is not easily achieved because of the separation of these data sets within the OpenSecrets database, though we note we were unable to access the bulk of data (which may provide a more interoperable data set). The LobbyView database (created by researchers at the Massachusetts Institute of Technology) also draws on US federal lobbying reports and presents opportunities to link lobbying activities to specific policies.^48^ It has previously been used to analyze US lobbying of the WHO.^14^ This could help to further our comparisons of patterns of political activities—are the same companies that spend the most on lobbying likewise the largest political donors or does this differ? Are some companies or trade associations only active in one or the other domain? Is there a correlation between the amount spent on lobbying and political donations at the level of individual clients or industry sectors? A further step to advance data analysis would be the creation of more granular and nuanced classification schemes.^49^ For instance, we relied on two OpenSecrets categories as a proxy for UPFs, but some of the individual companies in those categories earn far more revenue than others from UPFs. A schema to identify and classify a UPF company or other attributes relevant to public health would advance our capacity to analyze the health impacts of diverse commercial actors. Likewise, companies and their parent or holding companies are not always linked in the data set (for example, Hay Island Holdings is the holding company of Swisher International, but they are not connected in the OpenSecrets database). This speaks to the broader challenge of researching and monitoring companies (often with complex ownership structures) and trying to link information across multiple and varying databases.

Although many of the largest companies selling harmful products are embedded transnationally, data describing corporate–governmental lobbying are collected in line with mandated requirements and therefore vary across jurisdictions within and across countries. Resulting data sets are often dissimilar in granularity and completeness and may use different definitions and names. This makes constructing a global minimum data set of lobbying activity challenging.^32^ The Comparative Agendas Project provides examples of how diverse data sources can be linked and organized to create a commonly coded database across multiple country contexts.^50^ Further studies comparing the similarities and differences in lobbying or other political practices can help to understand the drivers and patterns of corporate political activity in different contexts.

## Conclusions

By comparing the lobbying practices of four different industry sectors, this study has helped to reveal the differences and similarities in how four industries engage in political activities. By building a better understanding of the strategies each industry sector uses, public health advocates may be better prepared to challenge the political opposition that these industries often present to public health policies.^51^

## Conflict of Interest Disclosures

J.L.‐N. is the recipient of a fellowship to research the CDoHs from the Victorian Health Promotion Foundation.
